# Supplementary material for: Profilin 1 deficiency drives mitotic defects and reduces genome stability
Source: Commun Biol. 2023 Jan 4;6:9. doi: 10.1038/s42003-022-04392-8 (PMC9813376; doi:10.1038/s42003-022-04392-8)
Supplement: Supplementary file 2 — Supplementary Information [file 42003_2022_4392_MOESM2_ESM.pdf]

Supplementary Information for:

Title

Profilin 1 deficiency drives mitotic defects and reduces genome stability

Authors

Federica Scotto di Carlo[1], Sharon Russo[1,2], Francesc Muyas[3], Maria Mangini[4], Lorenza Garribba[5], Laura Pazzaglia[6], Rita Genesio[7], Flavia Biamonte[8,9], Anna Chiara De Luca[4], Stefano Santaguida[5,10], Katia Scotlandi[6], Isidro Cortés-Ciriano[3,*], Fernando Gianfrancesco[1,*]

Affiliations

[1]Institute of Genetics and Biophysics "Adriano Buzzati-Traverso" (IGB), National Research Council of Italy (CNR), Naples, Italy;

[2]Department of Environmental, Biological and Pharmaceutical Sciences and Technologies (DiSTABiF), University of Campania Luigi Vanvitelli, Caserta, Italy;

[3]European Molecular Biology Laboratory, European Bioinformatics Institute, Wellcome Genome Campus, Hinxton, UK;

[4]Institute for Experimental Endocrinology and Oncology, "G. Salvatore" (IEOS), National Research Council of Italy (CNR), Naples, Italy;

[5]Department of Experimental Oncology at IEO, European Institute of Oncology IRCCS, Milan, Italy;

[6]IRCCS Istituto Ortopedico Rizzoli, Laboratory of Experimental Oncology, Bologna, Italy;

[7]Department of Molecular Medicine and Medical Biotechnology, University of Naples Federico II, Naples, Italy.

[8]Department of Experimental and Clinical Medicine, Magna Graecia University, Catanzaro, Italy;

[9]Center of Interdepartmental Services (CIS), Magna Graecia University, Catanzaro, Italy;

[10]Department of Oncology and Hemato-Oncology, University of Milan, Milan, Italy.

[*]Address correspondence to: icortes@ebi.ac.uk; fernando.gianfrancesco@igb.cnr.it

Supplementary Fig. 1

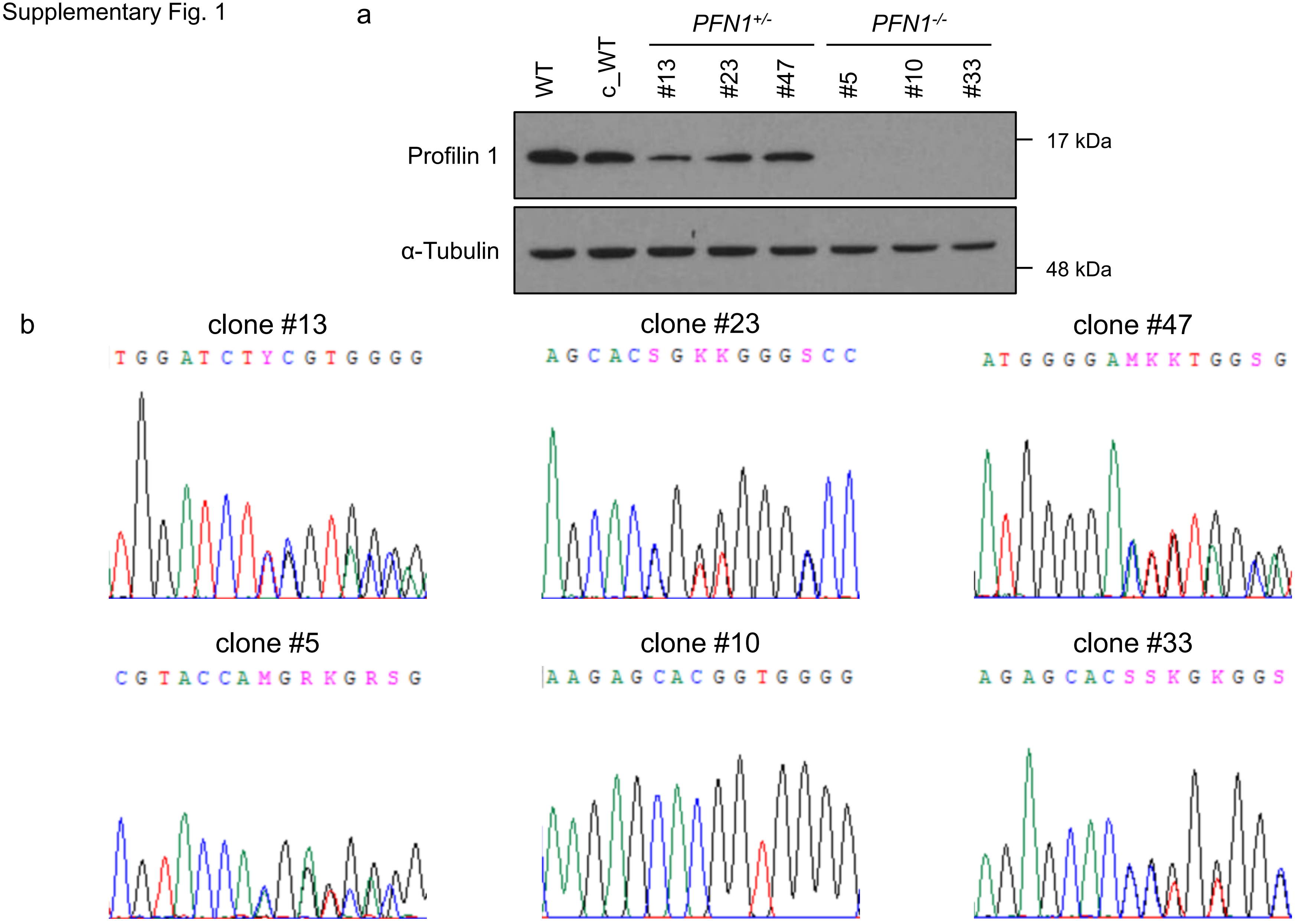

Supplementary Fig. 1: (a) Western blot analysis of Profilin 1 expression in 3 *PFN1*$^{+/-}$ and 3 *PFN1*$^{-/-}$ RPE1 clones compared with wild type parental cells (WT) and cells subjected to Cas9/gRNA transfection but resulted wild type for *PFN1* (c_WT); **(b)** Sanger sequencing results showing the CRISPR-Cas9-induced INDELs in the indicated RPE1 clones.

Supplementary Fig. 2

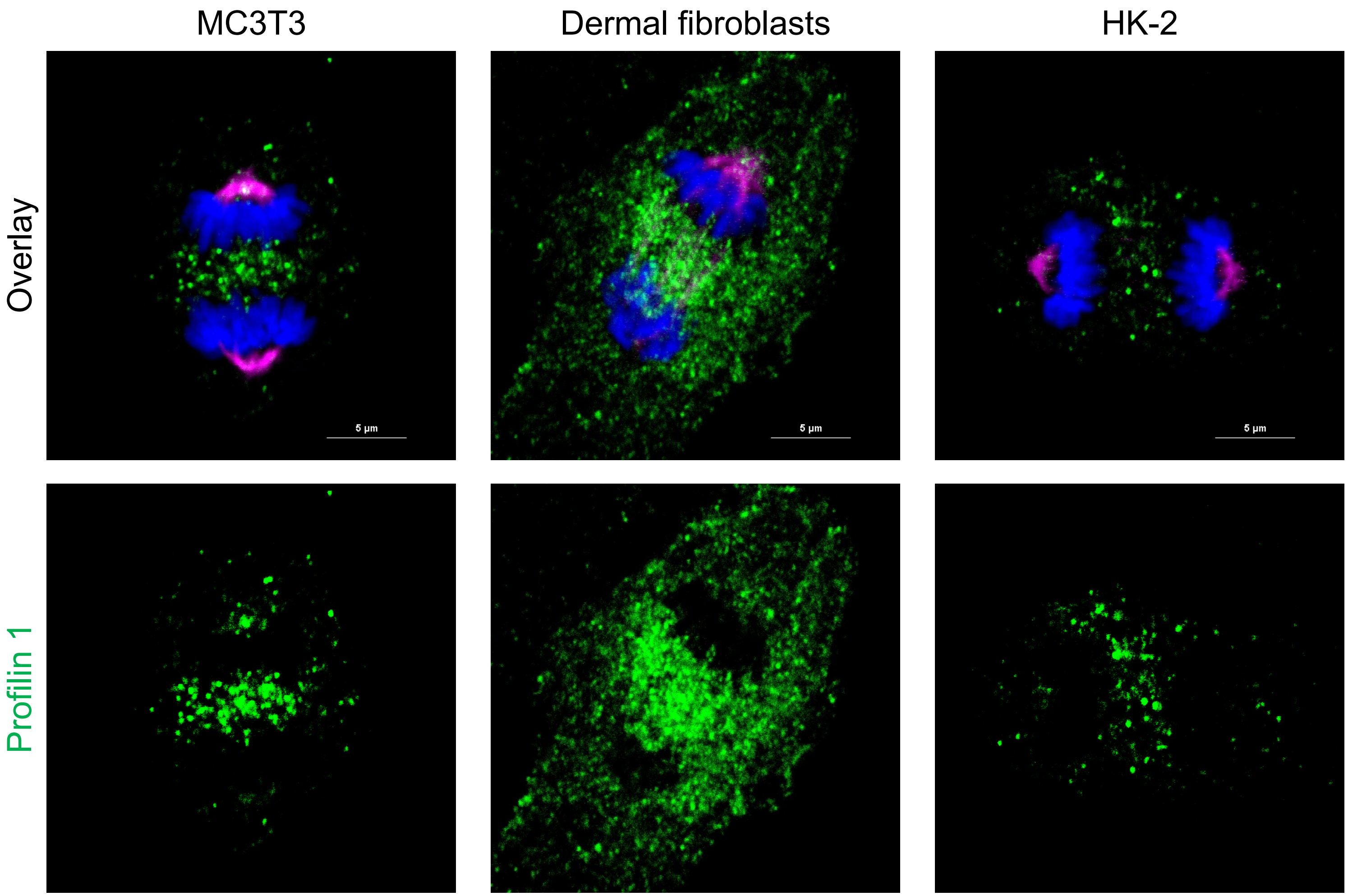

Supplementary Fig. 2: MC3T3, human dermal fibroblasts, and HK-2 cells at anaphase stained for DNA (Hoechst 33342, blue), alpha-tubulin (magenta) and Profilin 1 (green); scale bars 5 µm. The brightness for the green colour has been enhanced in the panels showing Profilin 1; original colour is shown in the overlay panels.

Supplementary Fig. 3

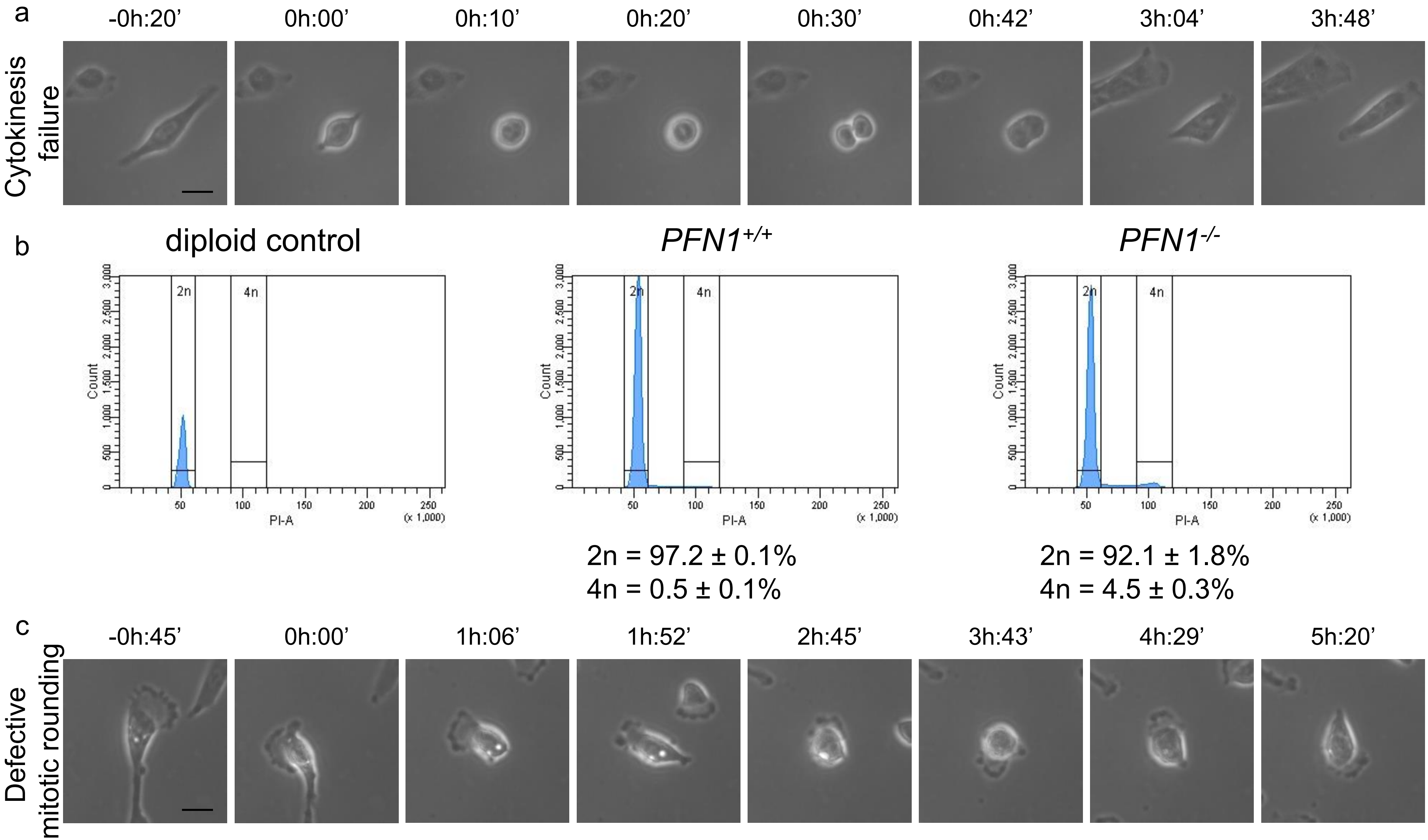

Supplementary Fig. 3: (a) Still images from time-lapse phase contrast videos of *PFN1*-KO RPE1 cells failing cytokinesis. **(b)** Representative images of propidium iodide incorporation in human PBMCs (diploid control) and serum-starved WT and $PFN1^{-/-}$ RPE1 cells. The percentage of interphase diploid (2n) and tetraploid (4n) cells is reported below the graphs; numbers indicate the mean ± standard deviation (*n* = 3 for WT and *n* = 4 for $PFN1^{-/-}$ RPE1 cells). **(c)** Still images from time-lapse phase contrast videos of *PFN1*-KO RPE1 cells undergoing incomplete mitotic rounding; scale bars 25 μm. Time stamps indicate elapsed time in hours:minutes. Images of wild type mitoses are illustrated in Figure 3.

Supplementary Fig. 4

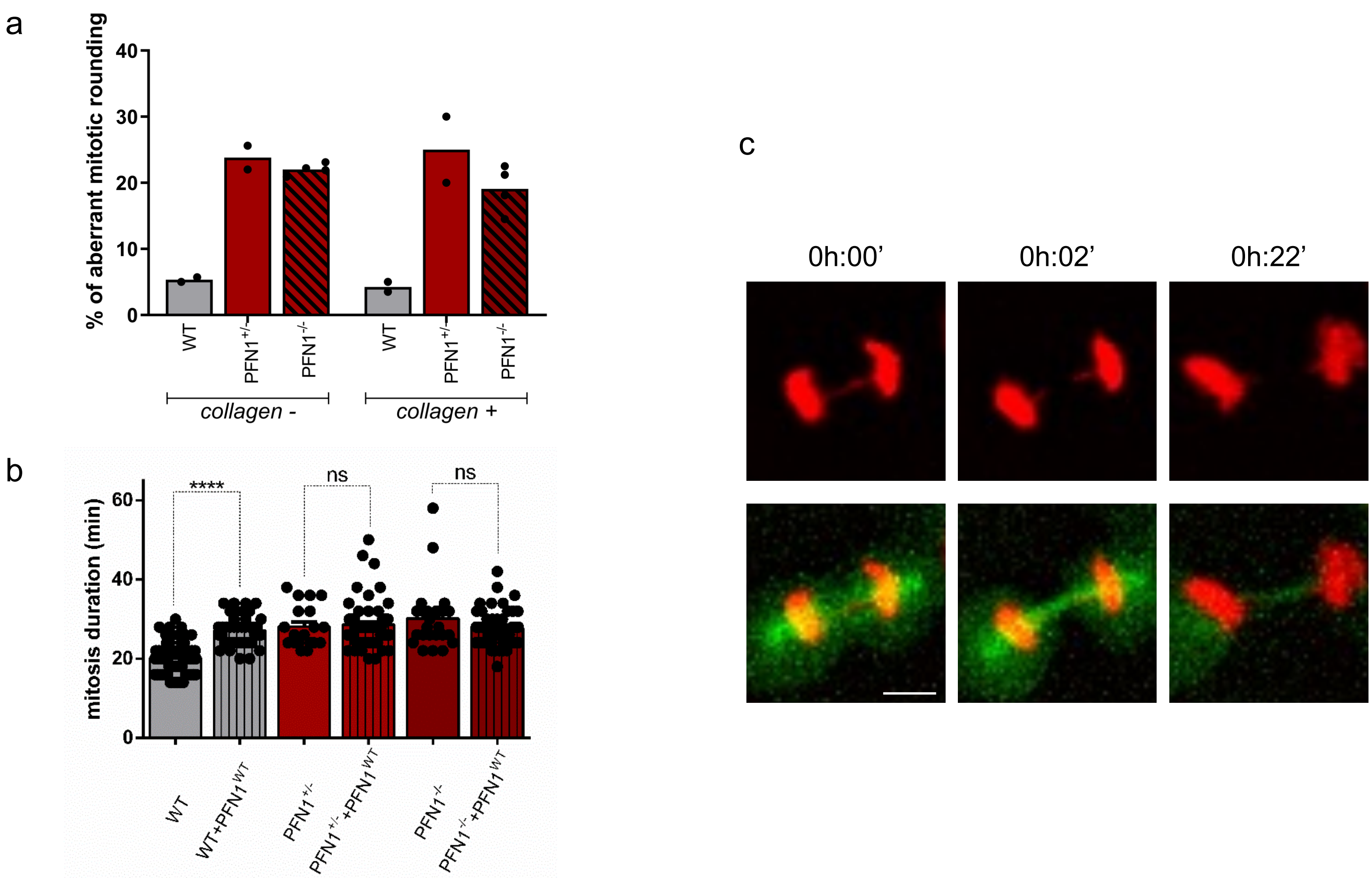

Supplementary Fig. 4: (a) Quantification (%) of non-sperichal metaphase cells in WT, *PFN1*$^{+/-}$, and *PFN1*$^{-/-}$ RPE1 cells after thymidine-nocodazole block. Bar graph shows the mean; dots represent individual experiments (*n* = 206 WT, 260 *PFN1*$^{+/-}$, and 139 *PFN1*$^{-/-}$ RPE1 cells on uncoated coverslips, and 244 WT, 238 *PFN1*$^{+/-}$, and 177 *PFN1*$^{-/-}$ RPE1 cells on collagen-coated coverslips; range of number of cells used for each individual experiment: 99-107 WT, 89-171 *PFN1*$^{+/-}$, 67-121 *PFN1*$^{-/-}$ on uncoated coverslips; 100-144 WT, 102-136 *PFN1*$^{+/-}$, 83-151 *PFN1*$^{-/-}$ on collagen-coated coverslips). **(b)** Time taken from start (cell rounding) to end (cytokinesis) of mitosis; data are shown as mean ± s.e.m.; (*n* mitoses = 77 WT, 42 WT+PFN1^{WT}, 19 PFN1$^{+/-}$, 64 PFN1$^{+/-}$+PFN1^{WT}, 25 PFN1$^{-/-}$, and 45 PFN1$^{-/-}$+PFN1^{WT} RPE1 cells). Data were analysed by Two-way ANOVA. **(c)** Fluorescence time-lapse microscopy of a *PFN1*-KO RPE1 cell undergoing mitosis, producing a chromosome bridge that breaks in telophase. Scale bar 10 µm. DNA is shown in red (H2B-mCherry) and microtubules in green (EGFP-Tubulin). Time stamps indicate elapsed time in hours:minutes.

Supplementary Fig. 5

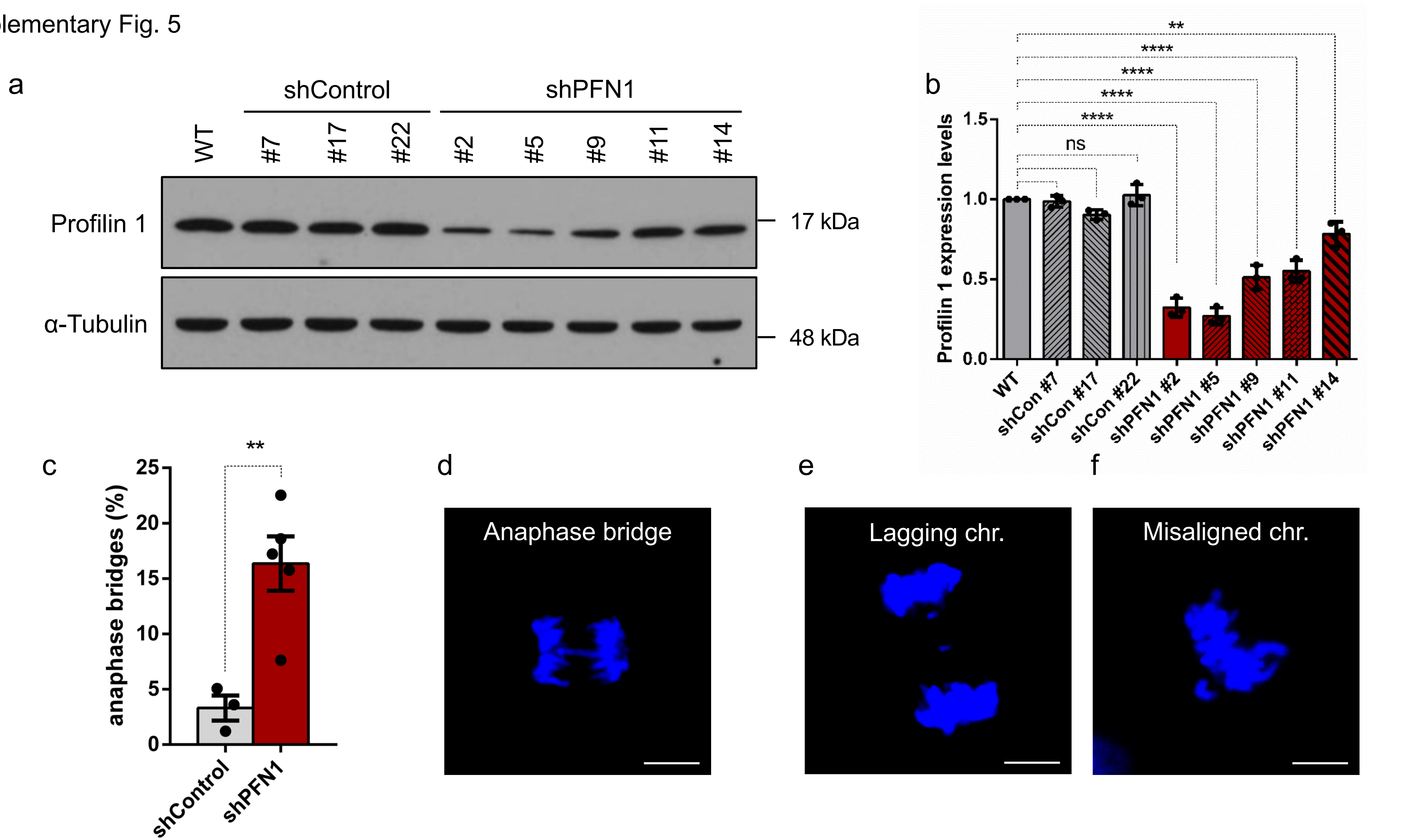

Supplementary Fig. 5: (a) Western blot analysis of Profilin 1 expression in 3 RPE1 clones expressing a non-target shRNA (shControl) and 5 RPE1 clones expressing the shPFN1. **(b)** Bar graph of band intensity quantification is shown (*n* = 3 independent experiments; ****P < 0,0001, **P = 0,0013). Ordinary one-way ANOVA performed. **(c)** The bar graph shows the quantification of anaphase bridges in RPE1 cells with and without *PFN1* knockdown; bars indicate mean ± s.e.m., dots represent the mean percentage of two independent experiments (*n* = 3 biological replicates for shControl, 5 for shPFN1 cells; scoring a total of 183 control and 307 knockdown mitoses; range of number of cells used for each individual experiment: 25-41 shControl, 21-48 shPFN1); ***P* = 0,0041. One-tailed unpaired Student's t-tests performed. **(d)** Confocal image of Hoechst-stained *PFN1* knockdown RPE1 cells showing an anaphase bridge. **(e,f)** Representative images of Hoechst-stained *PFN1* knockdown RPE1 cells with the indicated mitotic defects. Scale bars 5 μm.

Supplementary Fig. 6

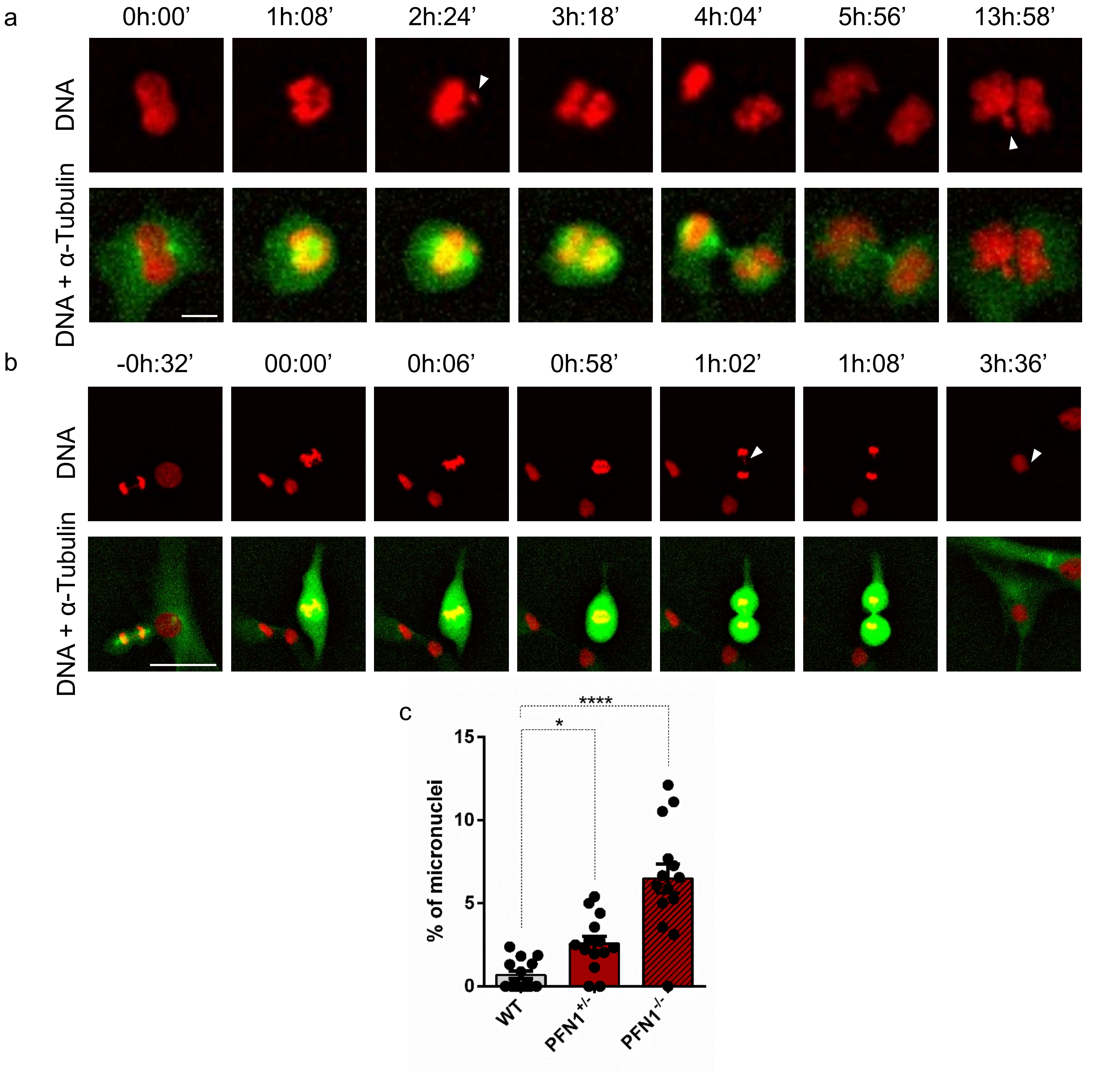

Supplementary Fig. 6: Fluorescence time-lapse microscopy of *PFN1*-KO RPE1 cells undergoing mitosis. **(a)** Mitotic KO cell showing a misaligned chromosome in metaphase (arrowhed), delayed anaphase onset, and cytokinesis failure resulting in one daughter cell containing two abnormal nuclei and one micronucleus (arrowhead). Scale bar 10 μm. **(b)** Mitotic KO cell starting metaphase without completely rounding up, delaying anaphase onset, and showing a lagging chromosome fragment (arrowhead) that results in an interphase micronucleus (arrowhead) in the daughter cell. Note the chromosome bridge formed in the mitotic cell on the left (first panel). Scale bar 50 μm. DNA is shown in red (H2B-mCherry) and microtubules in green (EGFP-Tubulin). Time stamps indicate elapsed time in hours:minutes. **(c)** Quantification (%) of micronucleated cells; data are shown as mean ± s.e.m.; dots represent the value of each field counted (*n* = 1082 WT, 840 $PFN1^{+/-}$, and 497 $PFN1^{-/-}$ cells; range of number of cells used for each individual experiment: 39-120 WT, 28-109 $PFN1^{+/-}$, 19-61 $PFN1^{-/-}$); **P* = 0,0484; *****P* < 0,0001. Ordinary one-way ANOVA performed.

Supplementary Fig. 7

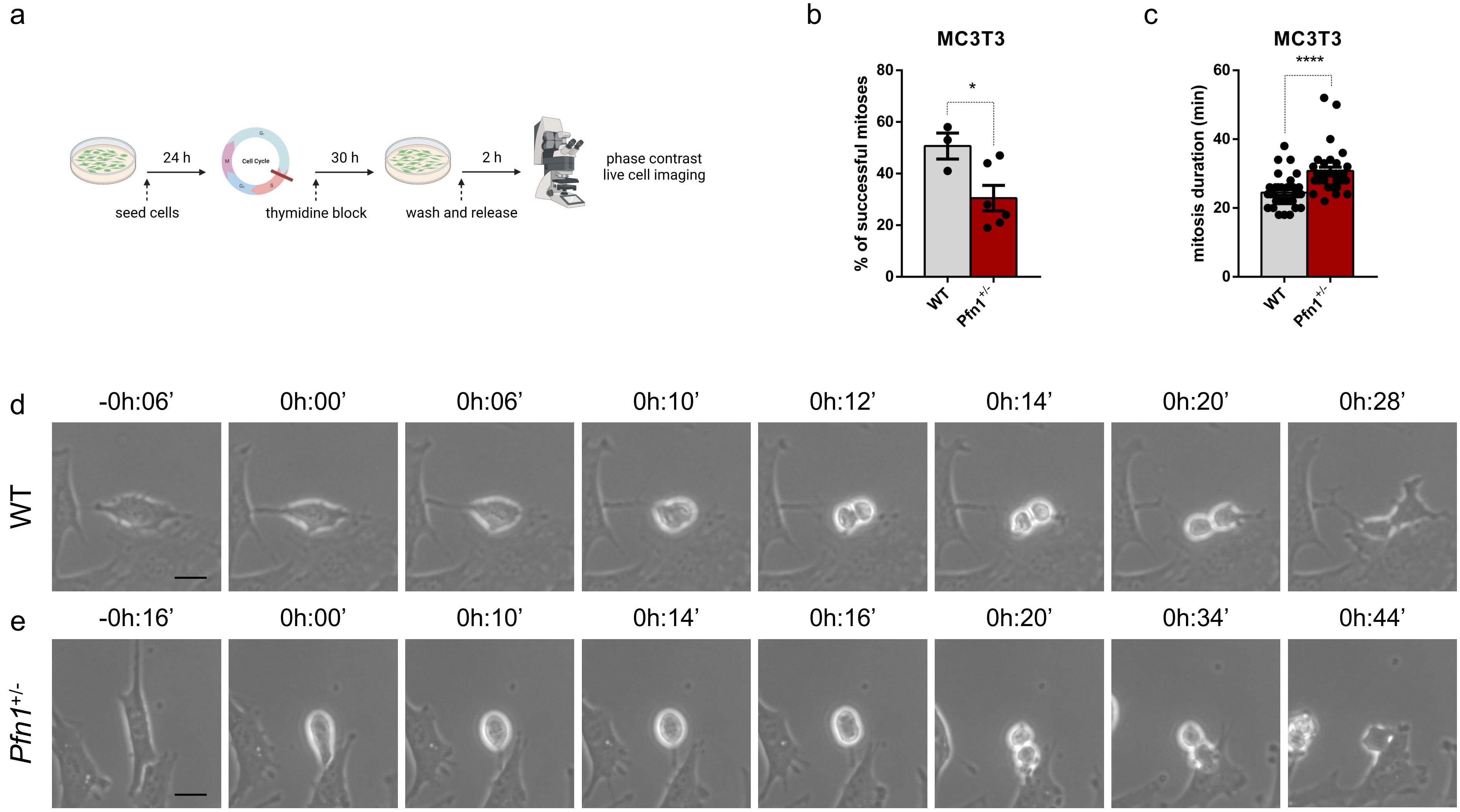

Supplementary Fig. 7: (a) Schematic of the experiment created in BioRender (https://biorender.com/) by F.S.d.C. as a registered user. **(b)** Percentage of cells able to complete mitosis following the round up; data are shown as mean ± s.e.m.; dots represent the mean for each experiment (n = 73 WT, 105 *Pfn1*$^{+/-}$ MC3T3 cells; range of number of cells used for each individual experiment: 17-30 WT, 14-25 *Pfn1*$^{+/-}$); *P = 0,0192. **(c)** Time taken from start (cell rounding) to end (cytokinesis) of mitosis; data are shown as mean ± s.e.m.; dots represent the duration of each cell division expressed in minutes (n mitoses = 33 WT, 32 *Pfn1*$^{+/-}$ MC3T3 cells); ****P < 0,0001. Data in **b** and **c** were analysed by one-tailed unpaired Student's *t*-tests. **(d)** Representative time-lapse phase contrast images (taken every 2 minutes) of WT and **(e)** *Pfn1*$^{+/-}$ MC3T3 cells; scale bars 25 μm. Time stamps indicate elapsed time in hours:minutes.

Supplementary Fig. 8

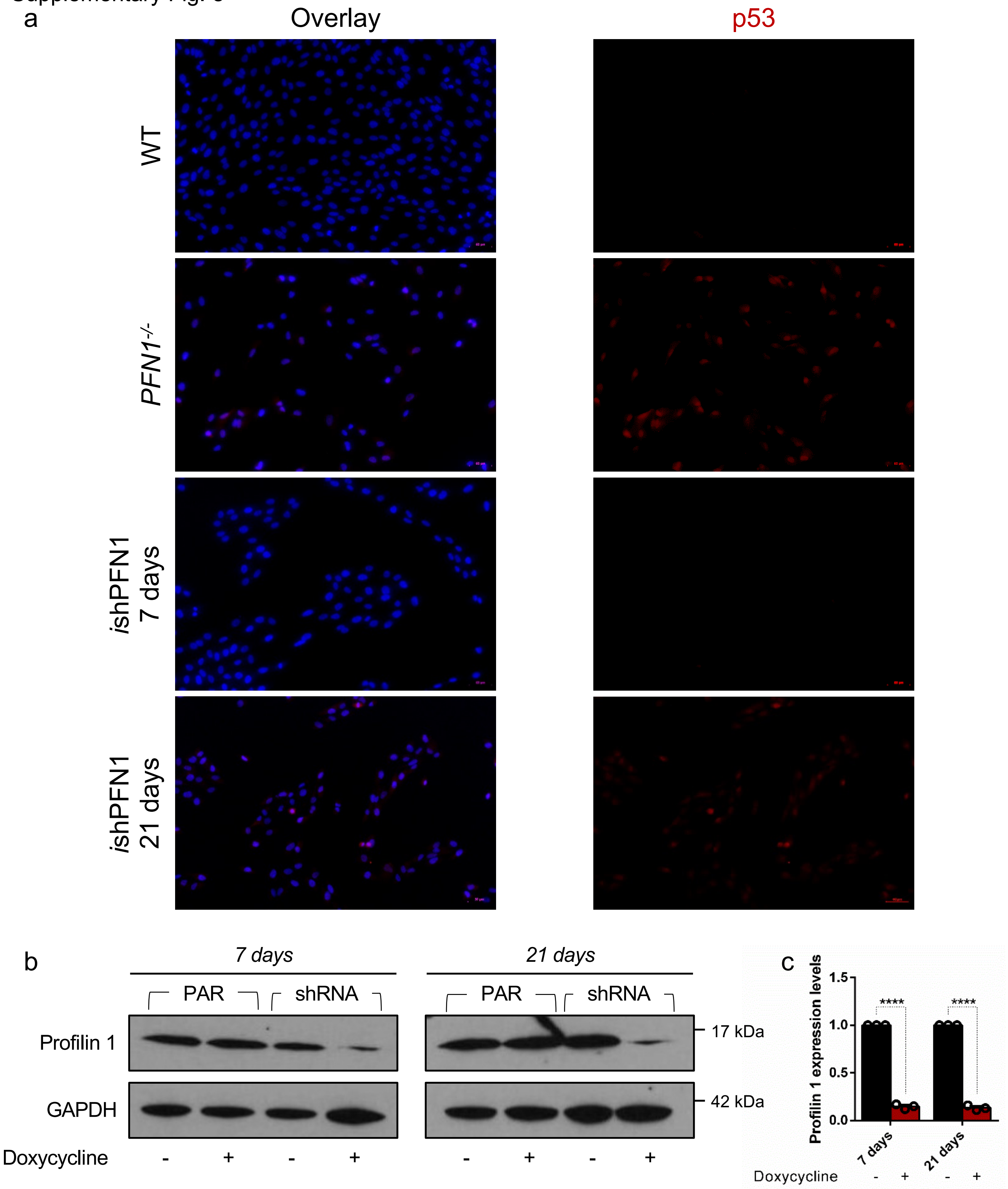

Supplementary Fig. 8: (a) Fluorescence miscropy images of p53 (red) in WT RPE1 cells compared with chronic (*PFN1*$^{-/-}$) and acute (*i*shPFN1) Profilin 1 inactivation. Merged panels include the blue channel (nuclei, Hoechst 33342). Scale bars 50 μm. **(b)** Western blotting showing Profilin 1 expression in parental (PAR) and shPFN1 (shRNA) RPE1 cells after 7 or 21 days of Doxycycline treatment. **(c)** Bar graph showing quantification of Profilin 1 in shRNA cells normalised to GAPDH; bars represent mean ± s.e.m.; dots represent the mean for each experiment (n = 3 from three independent protein extracts); ****P < 0.0001. Two-way ANOVA test performed.

Supplementary Fig. 9

a

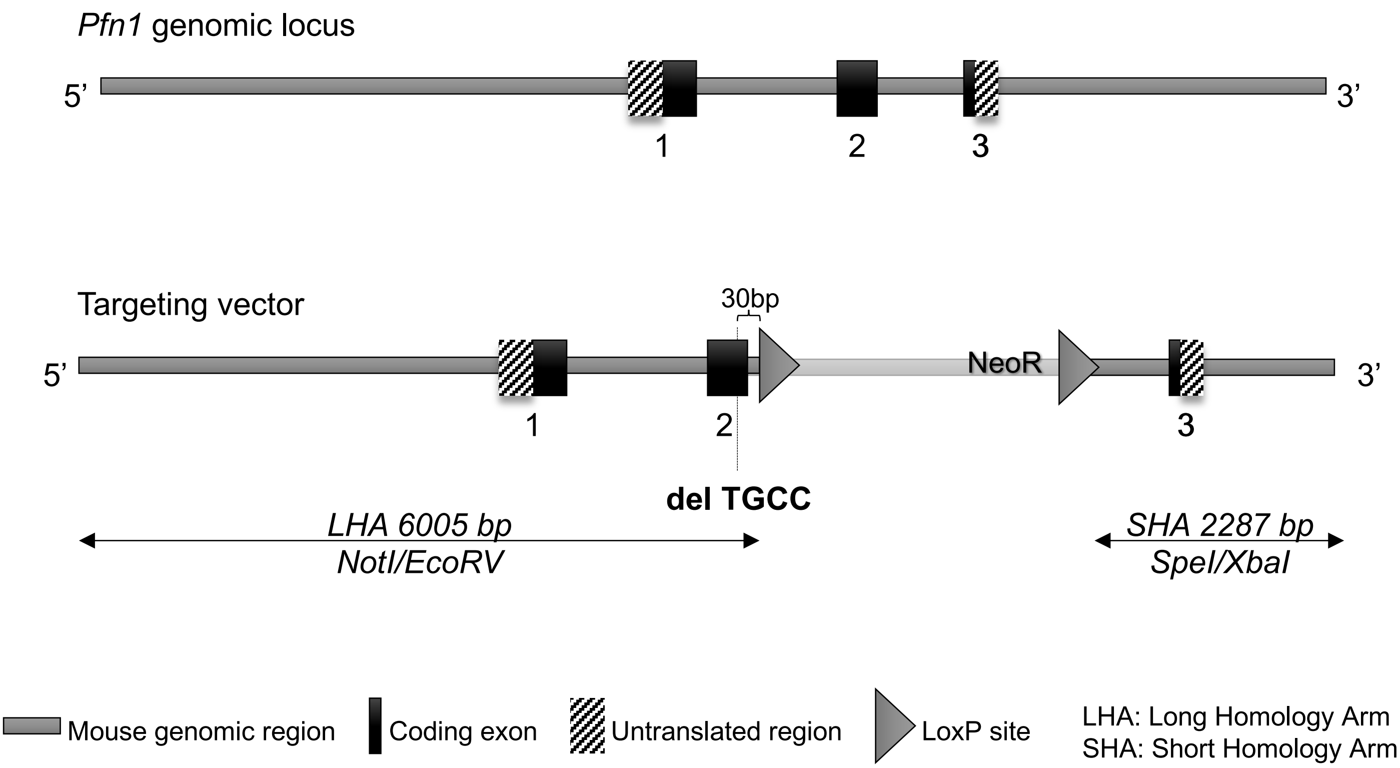

b

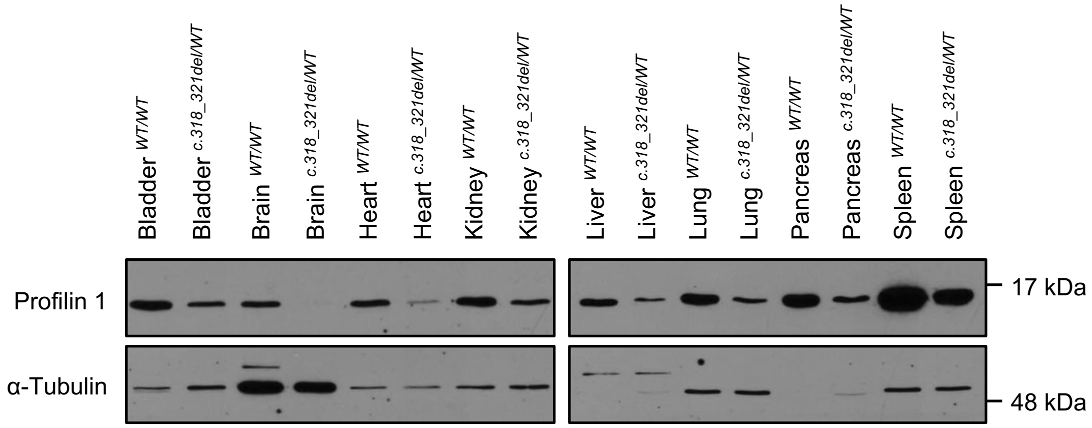

Supplementary Fig. 9: Generation of *Pfn1* c.318_321delTGCC constitutive knock-in mouse model. **(a)** Schematic representation of the wild type *Pfn1* locus and targeting strategy. The 4-bp deletion was inserted in the exon 2; LoxP-flanked Neo cassette was inserted in intron 2. **(b)** Western blotting analysis of Profilin 1 expression in the indicated mouse tissues; western blot of α-Tubulin represents internal controls. WT/WT indicates wild type tissues; c.318_321del/WT indicates tissues from *Pfn1* heterozygous knock-in animals.

Supplementary Fig. 10

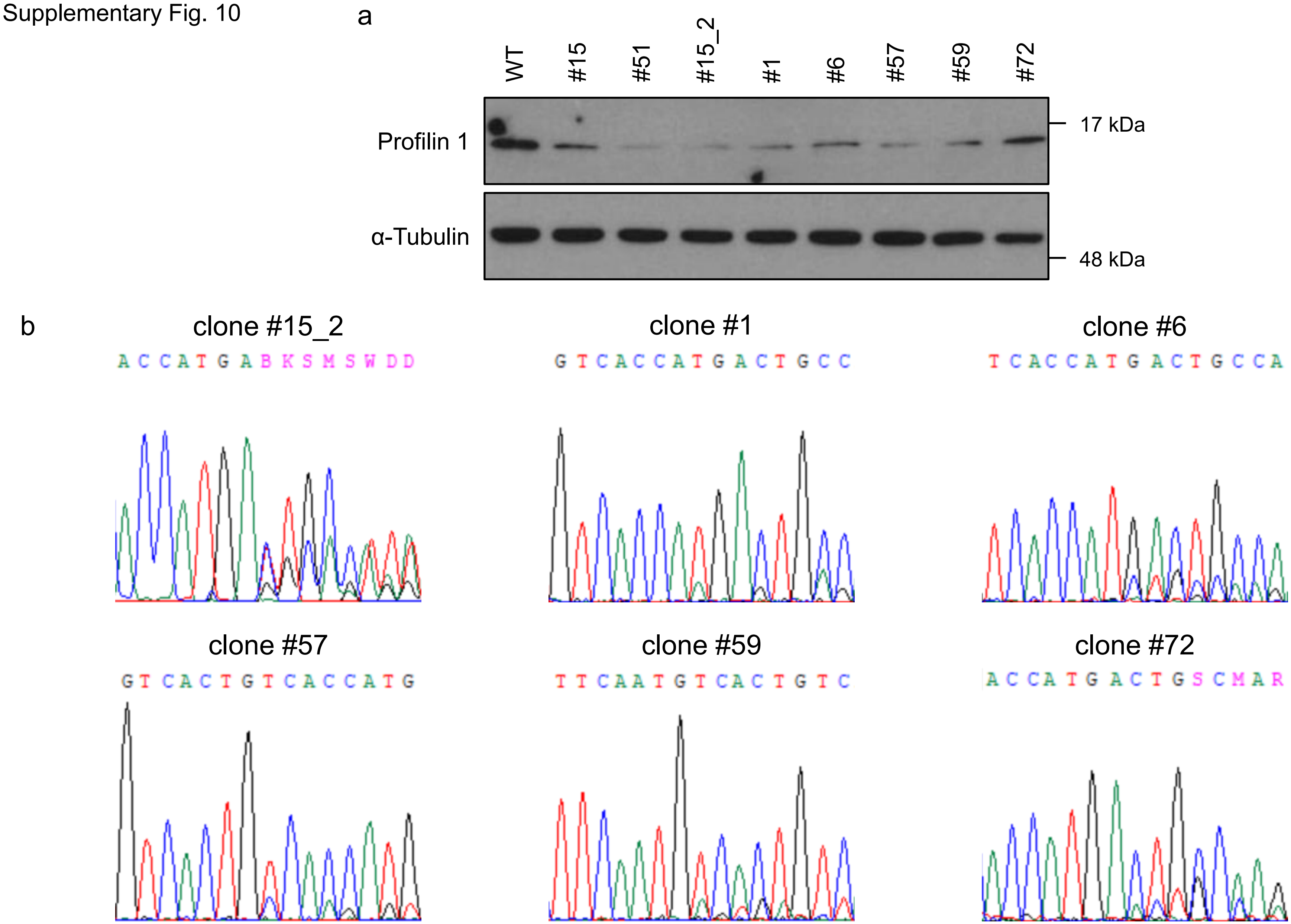

Supplementary Fig. 10: (a) Western blot analysis of Profilin 1 expression in 8 independent heterozygous *Pfn1*-KO MC3T3 extracts compared with WT cells; **(b)** Sanger sequencing results showing the CRISPR-Cas9-induced INDELs in the indicated MC3T3 clones. For the electropherogram plots of clones #15 and #51, see Ref. 26.

Supplementary Fig. 11

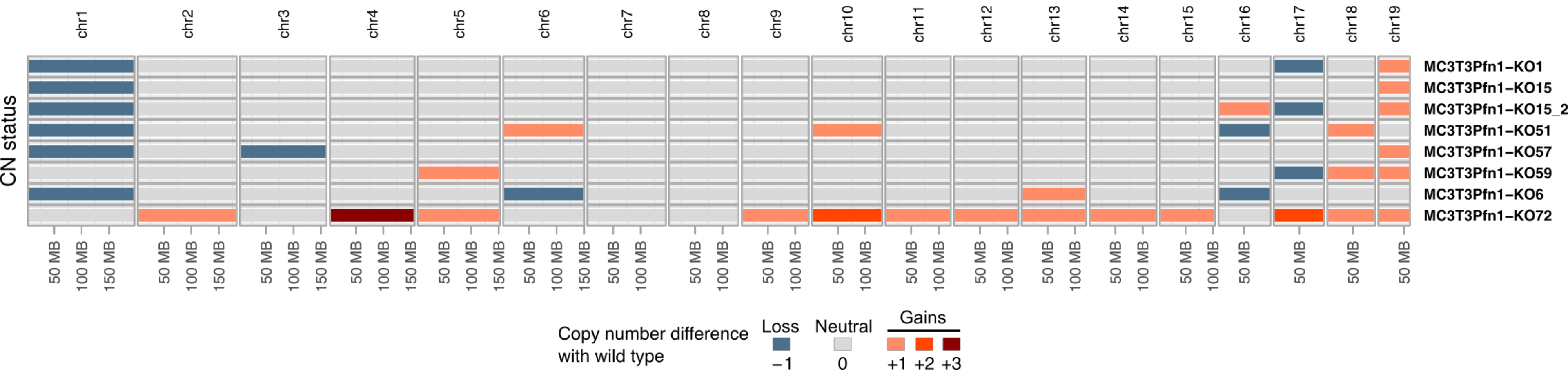

Supplementary Fig. 11: Chomosome-level copy-number profiles for the *Pfn1*$^{+/-}$ MC3T3 clones; the different colours indicate the levels of gains and losses.

Supplementary Fig. 12

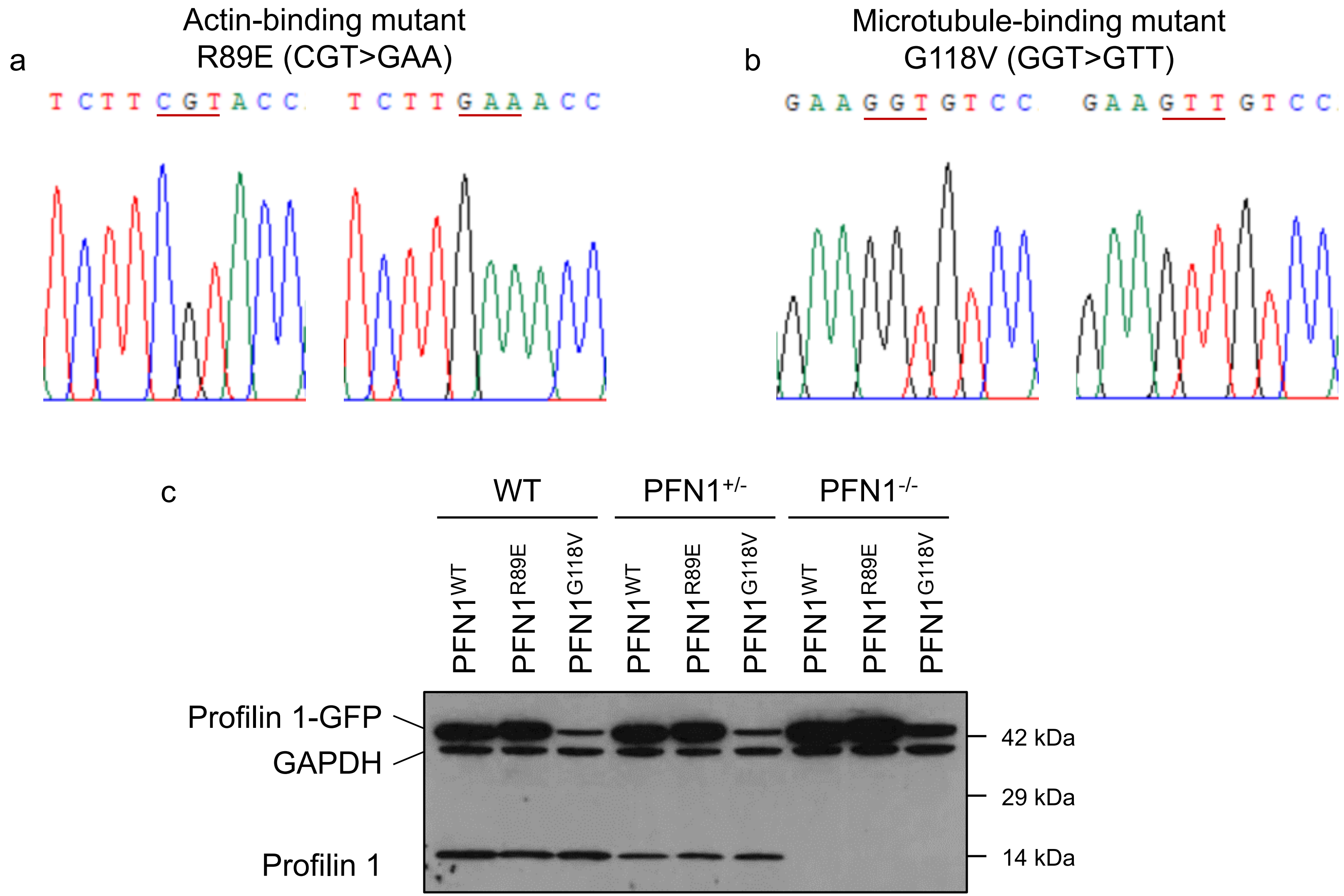

Supplementary Fig. 12: Sanger sequencing results showing **(a)** the R89E (CGT>GAA) and **(b)** the G118V (GGT>GTT) mutations inserted in the pLenti-PFN1-GFP plasmid. **(c)** Western blot analysis of Profilin 1 expression in WT, *PFN1*$^{+/-}$, and *PFN1*$^{-/-}$ RPE1 cells, each overexpressing either PFN1^{WT}-GFP, or PFN1^{R89E}-GFP, or PFN1^{G118V}-GFP. GAPDH was used as loading control. Note that the PFN1^{G118V} mutant is poorly stable and subjected to protein degradation.

Supplementary Fig. 13

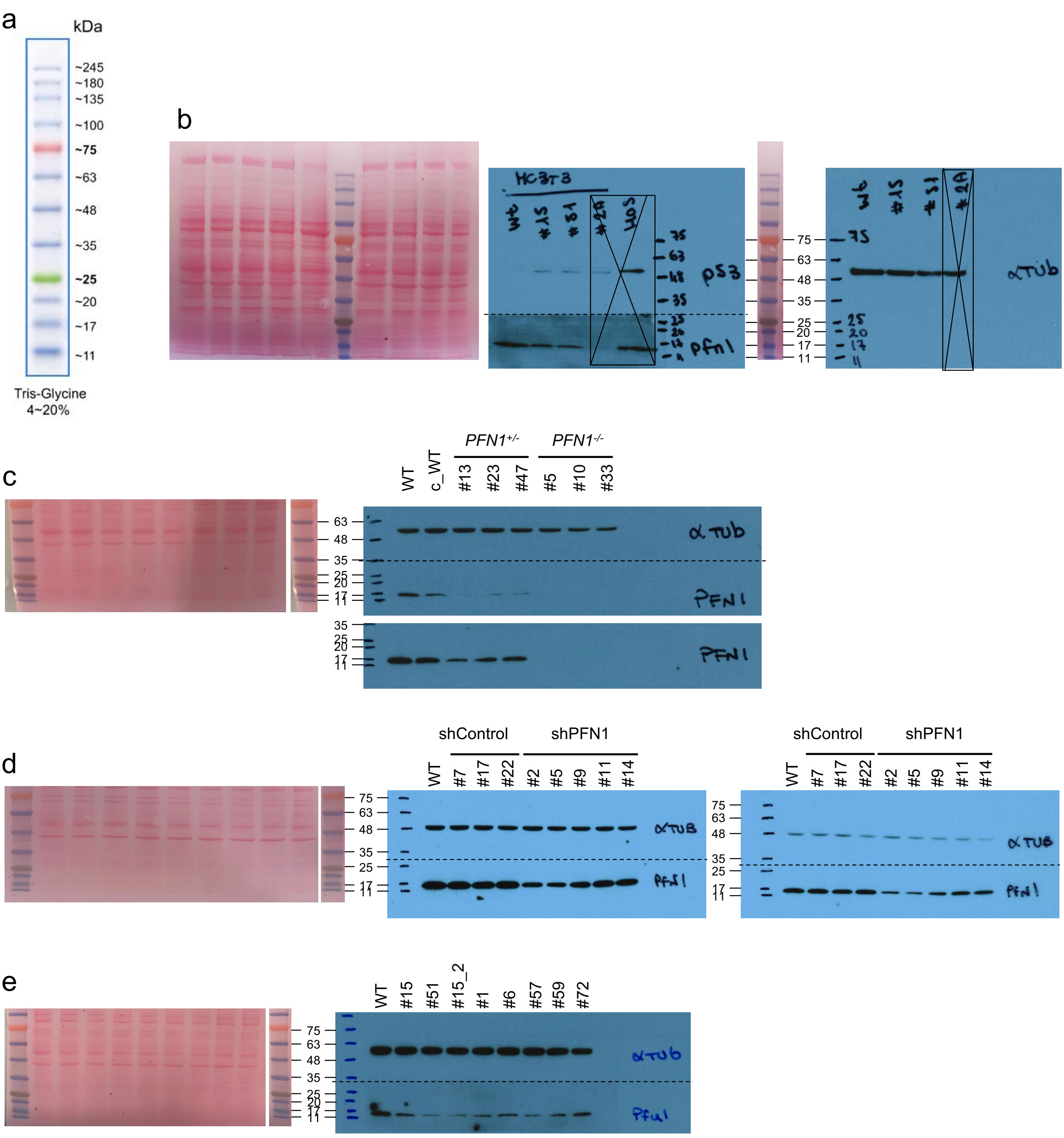

Supplementary Fig. 13: Uncropped and unedited blots. **(a)** Colorimetric molecular weight ladder used in the western blots shown in **b-e** (Opti-Protein XL Marker, abm #G266). **(b)** Original western blot for Figure 5i and relative ponceau red staining; due to similar molecular weight between the p53 and α-Tubulin proteins, the same amount of proteins was loaded two times on the same gel, separated by the ladder. Crosses indicate data not included in the current manuscript; hOS cells were used as positive control for p53 detection. **(c)** Original western blot and relative ponceau red staining for Supplementary Fig. 1; two different exposure times for Profilin 1 are shown. **(d)** Original western blot and relative ponceau red staining for Supplementary Fig. 5; two different exposure times are shown. **(e)** Original western blot and relative ponceau red staining for Supplementary Fig. 10. In **b-e**, dashed line indicates the different blots from the same gel incubated with the indicated antibodies.

Supplementary Fig. 14

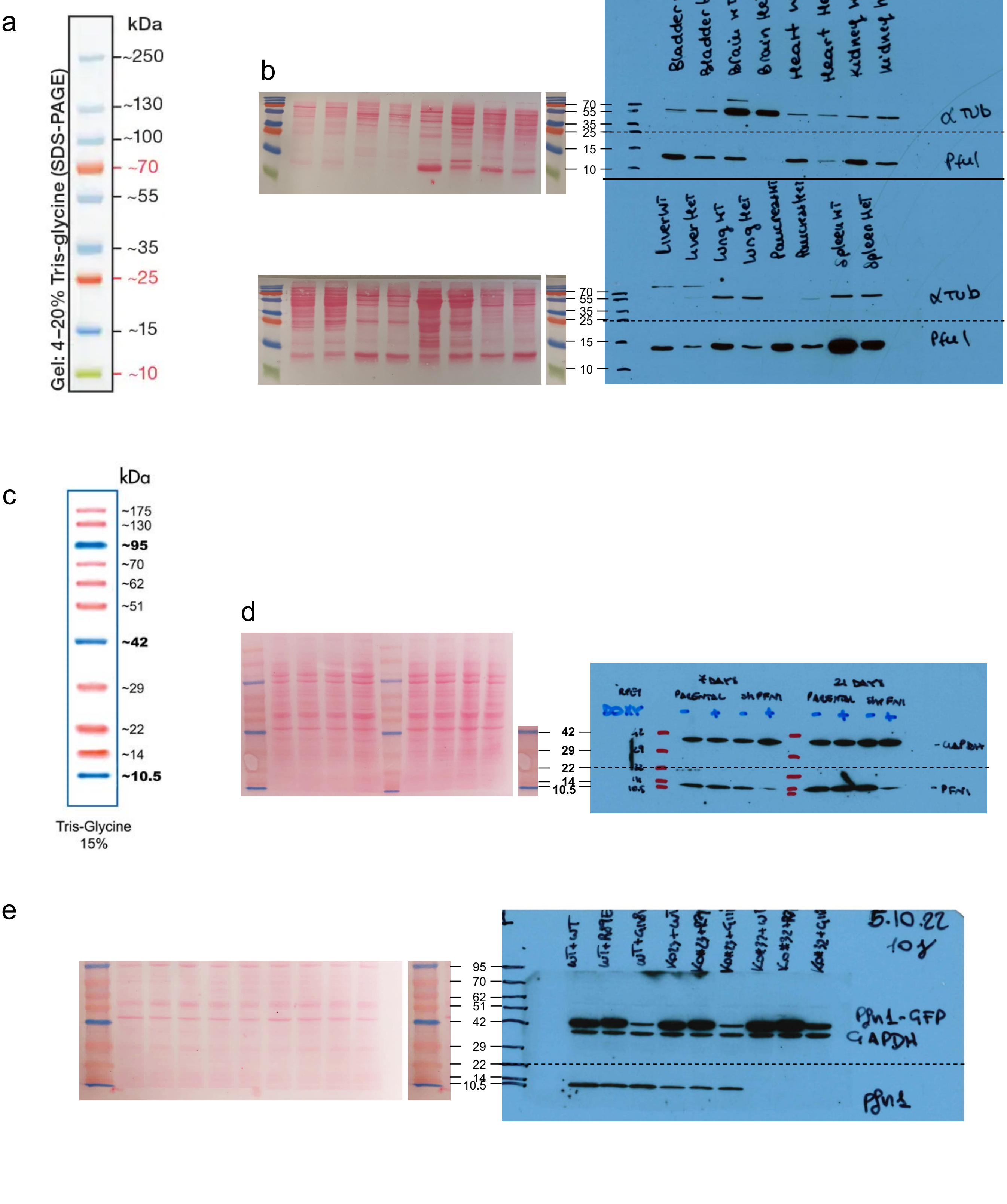

Supplementary Fig. 14: Uncropped and unedited blots. **(a)** Colorimetric molecular weight ladder used in the western blots shown in **b** (PageRuler™ Plus Prestained Protein Ladder, Thermo Scientific™ #26620). **(b)** Original western blot and relative ponceau red staining for Supplementary Fig. 9; solid line indicates two separate gels. **(c)** Colorimetric molecular weight ladder used in the western blots shown in **d,e** (Opti-Protein Marker, abm #G252). **(d)** Original western blot and relative ponceau red staining for Supplementary Fig. 8. **(e)** Original western blot and relative ponceau red staining for Supplementary Fig. 12. In **b,d,e**, dashed line indicates the different blots from the same gel incubated with the indicated antibodies.

Supplementary Table 1: Analysis of mitotic abnormalities in Profilin 1 knock-out RPE1 cells.

**RPE1 sample**	**Mitoses *(n)***	**Mitotic defects *(n; %)***	**Misaligned chromosomes *(n)***	**Multipolar spindles *(n)***	**Anaphase bridges *(n)***	**Lagging chromosomes *(n)***
Wild type	911	39; 4,3%	6	1	31	1
PFN1$^{+/-}$	825	234; 28,4%	29	38	148	19
PFN1$^{-/-}$	616	191; 31,0%	23	26	120	22
