# Supplementary material for: Profilin 1 deficiency drives mitotic defects and reduces genome stability
Source: Commun Biol. 2023 Jan 4;6:9. doi: 10.1038/s42003-022-04392-8 (PMC9813376; doi:10.1038/s42003-022-04392-8)
Supplement: Supplementary file 3 — Description of Additional Supplementary Files [file 42003_2022_4392_MOESM3_ESM.pdf]

Description of Additional Supplementary Files

File name: Supplementary Data 1
Description: Summary of the copy-number aberrations and whole genome doubling events identified in 14 OS/PDB patients.

File name: Supplementary Data 2
Description: Source data behind the graphs in the paper.

File name: Supplementary Data 3
Description: Individual accession numbers for RAW whole genome sequencing data of MC3T3 WT and Pfn1-KO clones deposited in the European Nucleotide Archive (ENA) at EMBL-EBI.

File name: Supplementary Movie 1.
Description: Time-lapse of WT RPE1 cells: Phase-contrast live cell imaging of WT RPE1 cells after thymidine synchronisation and release. The majority of mitoses occurred after 4-6 hours of recording (6-8 hours from release in thymidine-free medium). Images were acquired at 2 min intervals over a period of 17 hours and they are played back at 10 frames per second. Timestamp shows relative time in hours:minutes:seconds:milliseconds. Imaging was performed with a 20x objective.

File name: Supplementary Movie 2.
Description: Time-lapse of *PFN1*$^{+/-}$ RPE1 cells: Phase-contrast live cell imaging of heterozygous *PFN1*-KO RPE1 cells after thymidine synchronisation and release. The movie shows that after 8 hours (10 hours after the release) KO cells attempt to undergo cell division but struggle or fail to round up. Images were acquired at 2 min intervals over a period of 17 hours and they are played back at 10 frames per second. Timestamp shows relative time in hours:minutes:seconds:milliseconds. Imaging was performed with a 20x objective.

File name: Supplementary Movie 3.
Description
: Time-lapse of *PFN1*$^{-/-}$ RPE1 cells: Phase-contrast live cell imaging of homozygous *PFN1*-KO RPE1 cells after thymidine synchronisation and release. The movie shows that only few cells manage to undergo mitosis; after 10 hours (12 hours after the release) most KO cells struggle or fail to round up. Images were acquired at 2 min intervals over a period of 17 hours and they are played back at 10 frames per second. Timestamp shows relative time in hours:minutes:seconds:milliseconds. Imaging was performed with a 20x objective.

File name: Supplementary Movie 4.
Description: Time-lapse of WT H2B-mCherry/EGFP-Tubulin RPE1 cells: Fluorescence live cell imaging of WT RPE1 cells expressing H2B-mCherry and EGFP-Tubulin. Left panel: Merge overlay of DNA (H2B-mCherry, red) and microtubules (EGFP-Tubulin, green). Right panel: DNA (H2B-mCherry, red). Images were acquired at 2 min intervals over a period of 19 hours and they are played back at 10 frames per second. Timestamp shows relative time in hours:minutes:seconds. Images taken with 20x objective.

File name: Supplementary Movie 5.
Description: Time-lapse of *PFN1*$^{+/-}$ H2B-mCherry/EGFP-Tubulin RPE1 cells: Fluorescence live cell imaging of heterozygous *PFN1*-KO RPE1 cells expressing H2B-mCherry and EGFP-

Tubulin. Left panel: Merge overlay of DNA (H2B-mCherry, red) and microtubules (EGFP-Tubulin, green). Right panel: DNA (H2B-mCherry, red). The movie shows 3 abnormal mitoses: 1) chromosome misalignment on the metaphase plate at 7h:06'; 2) chromosome misalignment on the metaphase plate at 9h:16' and delayed anaphase onset; 3) delayed anaphase onset, formation of an anaphase bridge at 13h:42', and formation of daughter cells containing one micronucleus (15h:06'). Images were acquired at 2 min intervals over a period of 19 hours and they are played back at 10 frames per second. Timestamp shows relative time in hours:minutes:seconds. Images taken with 20x objective.

File name: Supplementary Movies 6,7
Description: Time-lapse of *PFN1*$^{-/-}$ H2B-mCherry/EGFP-Tubulin RPE1 cells: Fluorescence live cell imaging of homozygous *PFN1*-KO RPE1 cells expressing H2B-mCherry and EGFP-Tubulin. Left panel: Merge overlay of DNA (H2B-mCherry, red) and microtubules (EGFP-Tubulin, green). Right panel: DNA (H2B-mCherry, red). The movie 6 shows 3 abnormal mitoses: 1) formation of an anaphase bridge at 00h:10' and generation of a micronucleated daughter cell; 2) abnormal cell rounding at 01h:24', mitotic failure with the extrusion of chromosomes (or chromosome fragments) and formation of nuclear protrusions; 3) chromosome misalignment on the metaphase plate at 12h:00' and delayed anaphase onset. Note the dead cells floating in the culture medium. The movie 7 shows the formation of a chromosome bridge at 05h:24' undergoing breakage at 10h:52' and resulting in micronucleated daughter cells. Images were acquired at 2 min intervals over a period of 19 hours and they are played back at 10 frames per second. Timestamp shows relative time in hours:minutes:seconds. Images taken with 20x objective.

File name: Supplementary Movie 8.
Description: Time-lapse of WT MC3T3 cells: Phase-contrast live cell imaging of WT MC3T3 cells after thymidine synchronisation and release. The majority of mitoses occurred after 4-6 hours of recording (6-8 hours from release in thymidine-free medium); almost all cells divided. Images were acquired at 2 min intervals over a period of 17 hours and they are played back at 10 frames per second. Timestamp shows relative time in hours:minutes:seconds:milliseconds. Imaging was performed with a 20x objective.

File name: Supplementary Movie 9.
Description: Time-lapse of *Pfn1*$^{+/-}$ MC3T3 cells: Phase-contrast live cell imaging of heterozygous *Pfn1*$^{+/-}$ MC3T3 cells after thymidine synchronisation and release. The majority of mitoses occurred after 7-9 hours of recording (9-11 hours from release in thymidine-free medium); the movie shows that cells make one or more attempts before completely rounding up. Images were acquired at 2 min intervals over a period of 17 hours and they are played back at 10 frames per second. Timestamp shows relative time in hours:minutes:seconds:milliseconds. Imaging was performed with a 20x objective.

File name: Supplementary Movie 10.
Description: Time-lapse of WT MEFs: Phase-contrast live cell imaging of WT mouse embryonic fibroblasts. Images were acquired at 2 min intervals over a period of 16 hours and they are played back at 10 frames per second. Timestamp shows relative time in hours:minutes:seconds:milliseconds. Imaging was performed with a 20x objective.

File name: Supplementary Movie 11.
Description: Time-lapse of *Pfn1*$^{c.318\_321del/WT}$ MEFs: Phase-contrast live cell imaging of *Pfn1* heterozygous knock-in mouse embryonic fibroblasts. Yellow arrowheads point towards cells

undergoing cytokinesis failure and resulting in single daughter cells with ≥ 2 nuclei. White arrowheads point towards cells struggling or failing to round up. The asterisk indicates a cell giving rise to three daughter cells after division. Images were acquired at 2 min intervals over a period of 16 hours and they are played back at 10 frames per second. Timestamp shows relative time in hours:minutes:seconds:milliseconds. Imaging was performed with a 20x objective.
