# Supplementary material for: Profilin 1 deficiency drives mitotic defects and reduces genome stability
Source: Commun Biol. 2023 Jan 4;6:9. doi: 10.1038/s42003-022-04392-8 (PMC9813376; doi:10.1038/s42003-022-04392-8)
Supplement: Supplementary file 6 — Supplementary Data 3 [file 42003_2022_4392_MOESM6_ESM.docx]

**Captions for Supplementary Movies**

**Supplementary Movie 1. Time-lapse of WT RPE1 cells:** Phase-contrast live cell imaging of WT RPE1 cells after thymidine synchronisation and release. The majority of mitoses occurred after 4-6 hours of recording (6-8 hours from release in thymidine-free medium). Images were acquired at 2 min intervals over a period of 17 hours and they are played back at 10 frames per second. Timestamp shows relative time in hours:minutes:seconds:milliseconds. Imaging was performed with a 20x objective.

**Supplementary Movie 2. Time-lapse of *PFN1^+/-^* RPE1 cells:** Phase-contrast live cell imaging of heterozygous *PFN1*-KO RPE1 cells after thymidine synchronisation and release. The movie shows that after 8 hours (10 hours after the release) KO cells attempt to undergo cell division but struggle or fail to round up. Images were acquired at 2 min intervals over a period of 17 hours and they are played back at 10 frames per second. Timestamp shows relative time in hours:minutes:seconds:milliseconds. Imaging was performed with a 20x objective.

**Supplementary Movie 3. Time-lapse of *PFN1^-/-^* RPE1 cells:** Phase-contrast live cell imaging of homozygous *PFN1*-KO RPE1 cells after thymidine synchronisation and release. The movie shows that only few cells manage to undergo mitosis; after 10 hours (12 hours after the release) most KO cells struggle or fail to round up. Images were acquired at 2 min intervals over a period of 17 hours and they are played back at 10 frames per second. Timestamp shows relative time in hours:minutes:seconds:milliseconds. Imaging was performed with a 20x objective.

**Supplementary Movie 4. Time-lapse of WT H2B-mCherry/EGFP-Tubulin RPE1 cells:** Fluorescence live cell imaging of WT RPE1 cells expressing H2B-mCherry and EGFP-Tubulin. Left panel: Merge overlay of DNA (H2B-mCherry, red) and microtubules (EGFP-Tubulin, green). Right panel: DNA (H2B-mCherry, red). Images were acquired at 2 min intervals over a period of 19 hours and they are played back at 10 frames per second. Timestamp shows relative time in hours:minutes:seconds. Images taken with 20x objective.

**Supplementary Movie 5. Time-lapse of *PFN1^+/-^* H2B-mCherry/EGFP-Tubulin RPE1 cells:** Fluorescence live cell imaging of heterozygous *PFN1*-KO RPE1 cells expressing H2B-mCherry and EGFP-Tubulin. Left panel: Merge overlay of DNA (H2B-mCherry, red) and microtubules (EGFP-Tubulin, green). Right panel: DNA (H2B-mCherry, red). The movie shows 3 abnormal mitoses: 1) chromosome misalignment on the metaphase plate at 7h:06’; 2) chromosome misalignment on the metaphase plate at 9h:16’ and delayed anaphase onset; 3) delayed anaphase onset, formation of an anaphase bridge at 13h:42’, and formation of daughter cells containing one micronucleus (15h:06’). Images were acquired at 2 min intervals over a period of 19 hours and they are played back at 10 frames per second. Timestamp shows relative time in hours:minutes:seconds. Images taken with 20x objective.

**Supplementary Movies 6,7 Time-lapse of *PFN1^-/-^* H2B-mCherry/EGFP-Tubulin RPE1 cells:** Fluorescence live cell imaging of homozygous *PFN1*-KO RPE1 cells expressing H2B-mCherry and EGFP-Tubulin. Left panel: Merge overlay of DNA (H2B-mCherry, red) and microtubules (EGFP-Tubulin, green). Right panel: DNA (H2B-mCherry, red). The movie 6 shows 3 abnormal mitoses: 1) formation of an anaphase bridge at 00h:10’ and generation of a micronucleated daughter cell; 2) abnormal cell rounding at 01h:24’, mitotic failure with the extrusion of chromosomes (or chromosome fragments) and formation of nuclear protrusions; 3) chromosome misalignment on the metaphase plate at 12h:00’ and delayed anaphase onset. Note the dead cells floating in the culture medium. The movie 7 shows the formation of a chromosome bridge at 05h:24’ undergoing breakage at 10h:52’ and resulting in micronucleated daughter cells. Images were acquired at 2 min intervals over a period of 19 hours and they are played back at 10 frames per second. Timestamp shows relative time in hours:minutes:seconds. Images taken with 20x objective.

**Supplementary Movie 8. Time-lapse of WT MC3T3 cells:** Phase-contrast live cell imaging of WT MC3T3 cells after thymidine synchronisation and release. The majority of mitoses occurred after 4-6 hours of recording (6-8 hours from release in thymidine-free medium); almost all cells divided. Images were acquired at 2 min intervals over a period of 17 hours and they are played back at 10 frames per second. Timestamp shows relative time in hours:minutes:seconds:milliseconds. Imaging was performed with a 20x objective.

**Supplementary Movie 9. Time-lapse of *Pfn1^+/-^* MC3T3 cells:** Phase-contrast live cell imaging of heterozygous *Pfn1^+/-^* MC3T3 cells after thymidine synchronisation and release. The majority of mitoses occurred after 7-9 hours of recording (9-11 hours from release in thymidine-free medium); the movie shows that cells make one or more attempts before completely rounding up. Images were acquired at 2 min intervals over a period of 17 hours and they are played back at 10 frames per second. Timestamp shows relative time in hours:minutes:seconds:milliseconds. Imaging was performed with a 20x objective.

**Supplementary Movie 10. Time-lapse of WT MEFs:** Phase-contrast live cell imaging of WT mouse embryonic fibroblasts. Images were acquired at 2 min intervals over a period of 16 hours and they are played back at 10 frames per second. Timestamp shows relative time in hours:minutes:seconds:milliseconds. Imaging was performed with a 20x objective.

**Supplementary Movie 11. Time-lapse of *Pfn1^c.318_321del/WT^* MEFs:** Phase-contrast live cell imaging of *Pfn1* heterozygous knock-in mouse embryonic fibroblasts. Yellow arrowheads point towards cells undergoing cytokinesis failure and resulting in single daughter cells with ≥ 2 nuclei. White arrowheads point towards cells struggling or failing to round up. The asterisk indicates a cell giving rise to three daughter cells after division. Images were acquired at 2 min intervals over a period of 16 hours and they are played back at 10 frames per second. Timestamp shows relative time in hours:minutes:seconds:milliseconds. Imaging was performed with a 20x objective.
