# Supplementary material for: Profilin 1 deficiency drives mitotic defects and reduces genome stability
Source: Commun Biol. 2023 Jan 4;6:9. doi: 10.1038/s42003-022-04392-8 (PMC9813376; doi:10.1038/s42003-022-04392-8)
Supplement: Supplementary file 19 — Reporting Summary [file 42003_2022_4392_MOESM19_ESM.pdf]

nature portfolio

Corresponding author(s): Fernando Gianfrancesco

Last updated by author(s): Dec 2, 2022

Reporting Summary

Nature Portfolio wishes to improve the reproducibility of the work that we publish. This form provides structure for consistency and transparency in reporting. For further information on Nature Portfolio policies, see our Editorial Policies and the Editorial Policy Checklist.

Statistics

For all statistical analyses, confirm that the following items are present in the figure legend, table legend, main text, or Methods section.

n/a	Confirmed	
☐	☒	The exact sample size (*n*) for each experimental group/condition, given as a discrete number and unit of measurement
☐	☒	A statement on whether measurements were taken from distinct samples or whether the same sample was measured repeatedly
☐	☒	The statistical test(s) used AND whether they are one- or two-sided<br>*Only common tests should be described solely by name; describe more complex techniques in the Methods section.*
☒	☐	A description of all covariates tested
☒	☐	A description of any assumptions or corrections, such as tests of normality and adjustment for multiple comparisons
☐	☒	A full description of the statistical parameters including central tendency (e.g. means) or other basic estimates (e.g. regression coefficient) AND variation (e.g. standard deviation) or associated estimates of uncertainty (e.g. confidence intervals)
☐	☒	For null hypothesis testing, the test statistic (e.g. *F*, *t*, *r*) with confidence intervals, effect sizes, degrees of freedom and *P* value noted<br>*Give P values as exact values whenever suitable.*
☒	☐	For Bayesian analysis, information on the choice of priors and Markov chain Monte Carlo settings
☒	☐	For hierarchical and complex designs, identification of the appropriate level for tests and full reporting of outcomes
☒	☐	Estimates of effect sizes (e.g. Cohen's *d*, Pearson's *r*), indicating how they were calculated

Our web collection on statistics for biologists contains articles on many of the points above.

Software and code

Policy information about availability of computer code

Data collection	An Illumina HiSeq 4000 instrument was used to obtain raw whole-genome and whole-exome sequencing data.
Data analysis	Alignment: BWA-MEM, version 0.7.17-r1188;<br>Processing of aligned BAM reads: GATK, version 4.1.8.0;<br>Somatic SNV and indel calling: Mutect282, GATK, version 4.1.8.0; MuSE83, version 1.0rc; Strelka2, version 2.9.2;<br>Somatic CNA and ploidy purity: ascatNGS (for WGS); GATK, version 4.1.8.0 and FreeBayes90, version 1.3 (for WES);<br>Structural variants: Delly86, version 0.8.3; LUMPY, version 9–120.2.13; Manta, version 1.6.0; SvABA, version 1.1.3;<br>Statistical analysis: GraphPad Prism 6.0;<br>Microscopy image processing: NIS-Elements Advanced Research Analysis and ZEN pro software;<br>Western blot band intensity: ImageJ.

For manuscripts utilizing custom algorithms or software that are central to the research but not yet described in published literature, software must be made available to editors and reviewers. We strongly encourage code deposition in a community repository (e.g. GitHub). See the Nature Portfolio guidelines for submitting code & software for further information.

Data

Policy information about availability of data

All manuscripts must include a data availability statement. This statement should provide the following information, where applicable:

- Accession codes, unique identifiers, or web links for publicly available datasets
- A description of any restrictions on data availability
- For clinical datasets or third party data, please ensure that the statement adheres to our policy

All data used in this study are provided in the form of figures. Processed sequencing data are submitted as supplementary data file; raw data are available upon request.

Field-specific reporting

Please select the one below that is the best fit for your research. If you are not sure, read the appropriate sections before making your selection.

☒ Life sciences ☐ Behavioural & social sciences ☐ Ecological, evolutionary & environmental sciences

For a reference copy of the document with all sections, see nature.com/documents/nr-reporting-summary-flat.pdf

Life sciences study design

All studies must disclose on these points even when the disclosure is negative.

Sample size	Sample sizes were determined according to literature suggestions or previous experiments carried out in our laboratories.
Data exclusions	No data were excluded from the analyses presented in this study.
Replication	Most of the experiments were repeated at least three times with duplicate or triplicate of technical or biological replicates.
Randomization	N/A
Blinding	N/A

Reporting for specific materials, systems and methods

We require information from authors about some types of materials, experimental systems and methods used in many studies. Here, indicate whether each material, system or method listed is relevant to your study. If you are not sure if a list item applies to your research, read the appropriate section before selecting a response.

Materials & experimental systems

n/a	Involved in the study	
☐	☒	Antibodies
☐	☒	Eukaryotic cell lines
☒	☐	Palaeontology and archaeology
☐	☒	Animals and other organisms
☒	☐	Human research participants
☐	☒	Clinical data
☒	☐	Dual use research of concern

Methods

n/a	Involved in the study	
☒	☐	ChIP-seq
☐	☒	Flow cytometry
☒	☐	MRI-based neuroimaging

Antibodies

Antibodies used	Antibodies in western blotting experiments: rabbit anti-Profilin 1 (Novus Biologicals, NB200-162, 1:10’000 for human lysates; Invitrogen, PA5-17444, 1:5’000 for murine lysates); mouse anti-p53 (Cell Signaling, 2524, 1:1’000); mouse anti-α-Tubulin (Sigma, T6074; 1:15’000); mouse anti-GAPDH (SC-32233; 1:1’000). Antibodies in immunofluorescence experiments: rabbit anti-γ-H2AX (Cell Signaling, 2577, 1:400); mouse anti-p53 (Cell Signaling, 2524, 1:2’000); mouse anti-α-Tubulin (Sigma, T6074; 1:500); rabbit anti-Profilin 1 (Novus Biologicals, NB200-162, 1:100); mouse anti-Aurora B (BD Transduction Laboratories, 611082, 1:100); phospho-Histone H3 (Cell Signaling, 53348; 1:1600) .
Validation	Validation was provided by the manufacturer's websites and appropriate controls were also used in our experiments.

Eukaryotic cell lines

Policy information about cell lines

Cell line source(s)	hTERT-RPE1 were gifted from Dr. Brunella Franco (Tigem, Naples, Italy); MC3T3, HK-2, and HEK293T were drawn from stock at Institute of Genetics and Biophysics (IGB-CNR, Naples, Italy). MEFs and calvaria-derived cells were obtained from mouse embryos and pups, respectively, using published protocols as indicated in the Methods. Human peripheral blood mononuclear cells (PBMCs) were isolated from a healthy donor by density-gradient centrifugation using Ficoll-Histopaque solution. Human dermal fibroblasts were previously isolated from a skin biopsy of a healthy donor and were drawn from stock at the biobank available at our Institute.
Authentication	The cell lines were not recently authenticated.
Mycoplasma contamination	All cell lines were tested for mycoplasma contamination (PCR Mycoplasma Detection Kit, Applied Biological Materials, G238) and were found to be negative.
Commonly misidentified lines (See ICLAC register)	N/A

Animals and other organisms

Policy information about studies involving animals; ARRIVE guidelines recommended for reporting animal research

Laboratory animals	Laboratory animals used in this study were 4-month-old mice maintained on a mixed genetic background (C57BL/6 and DBA/2). Embryos (E14.5) and pups (P2/3) were used for cell isolation, as described in the Methods.
Wild animals	N/A
Field-collected samples	N/A
Ethics oversight	Animals were handled in accordance with the authorization no. 125-2021-PR released by the Italian Ministry of Health and were approved by the Institute of Genetics and Biophysics (IGB) Institutional Animal Care and Use Committee (IACUC).

Note that full information on the approval of the study protocol must also be provided in the manuscript.

Clinical data

Policy information about clinical studies

All manuscripts should comply with the ICMJE guidelines for publication of clinical research and a completed CONSORT checklist must be included with all submissions.

Clinical trial registration	*Provide the trial registration number from ClinicalTrials.gov or an equivalent agency.*
Study protocol	*Note where the full trial protocol can be accessed OR if not available, explain why.*
Data collection	*Describe the settings and locales of data collection, noting the time periods of recruitment and data collection.*
Outcomes	*Describe how you pre-defined primary and secondary outcome measures and how you assessed these measures.*

Flow Cytometry

Plots

Confirm that:

☒ The axis labels state the marker and fluorochrome used (e.g. CD4-FITC).

☒ The axis scales are clearly visible. Include numbers along axes only for bottom left plot of group (a 'group' is an analysis of identical markers).

☒ All plots are contour plots with outliers or pseudocolor plots.

☒ A numerical value for number of cells or percentage (with statistics) is provided.

Methodology

Sample preparation	After serum starvation, the RPE1 cells were harvested and washed with cold PBS. Cells were fixed in ice-cold 100% methanol and incubated overnight at -20 °C. For FACS analysis, fixed cells were resuspended in PBS containing 0.2 mg/ml RNase, and incubated for 1 h at 37 °C. Then, cells were stained with 50 mg/ml propidium iodide for 30 min.
Instrument	Becton Dickinson FACSAria
Software	BD software

Cell population abundance	N/A
Gating strategy	PBMCs were used as a diploid internal standard to accurately identify the G0/G1 diploid peak position.

☐ Tick this box to confirm that a figure exemplifying the gating strategy is provided in the Supplementary Information.
